# The synergistic antitumor effects of psoralidin and cisplatin in gastric cancer by inducing ACSL4-mediated ferroptosis

**DOI:** 10.1186/s41065-025-00591-5

**Published:** 2025-11-04

**Authors:** Ling Yao, Jinhua Yan, Lihong Gan, Li Zheng, Peng Liu, Ling Lei, Yaqin Huang

**Affiliations:** 1 Department of Gastroenterology, The First Hospital of Nanchang, 128 Xiangshan North Road, Nanchang, Jiangxi Province 330008 China; 2 Department of Hematology, The First Hospital of Nanchang, Nanchang, Jiangxi 330008 China

**Keywords:** Psoralidin, Gastric cancer, Cisplatin, Ferroptosis, Toxic effects

## Abstract

**Objective:**

Cisplatin (DDP) is the major chemotherapeutic drug used to treat gastric cancer (GC). However, DDP-associated side effects and resistance chemoresistance have limited its clinical application. Psoralidin (PSO) is the main extract of *Psoralea corylifolia* and has antitumor effects. The present study is designed to investigate the antitumor functions and mechanisms of PSO and DDP in GC.

**Methods:**

GC cells (HGC-27 and MKN-45 cells) were treated with PSO (2.5 to 120 µM) and/or DDP. A CCK-8 assay, colony formation assay, and EdU staining were used to test cell proliferation. Cell migration and invasion were tested via a transwell assay. An in vivo assay in nude mice was carried out to analyze the influence of PSO and DDP on tumor growth. H&E staining was conducted to test the histopathological changes of organs and tumor tissues. Ferroptosis-associated indicators, including GSH, MDA, Fe^2+^ levels, were examined. Western *blotting* was conducted to determine the profiles of ACSL4, GPX4, AIFM2, and SLC7A11.

**Results:**

PSO impeded GC cell proliferation, migration, invasion, and growth in vivo. PSO exhibited no significant toxic effects on organs and mitigated DDP-mediated liver and kidney injuries. The combination of PSO and DDP exhibited enhanced inhibitory functions. PSO and DDP can significantly promote GC cell ferroptosis. Moreover, PSO promoted ACSL4 expression and suppressed GPX4, AIFM2, and SLC7A11.

**Conclusion:**

The combination of PSO and DDP has synergistic antitumor effects on GC cells by inducing ACSL4-mediated ferroptosis. PSO may serve as a nontoxic adjuvant to enhance DDP’s efficacy and reduce side effects in GC.

**Graphical abstract:**

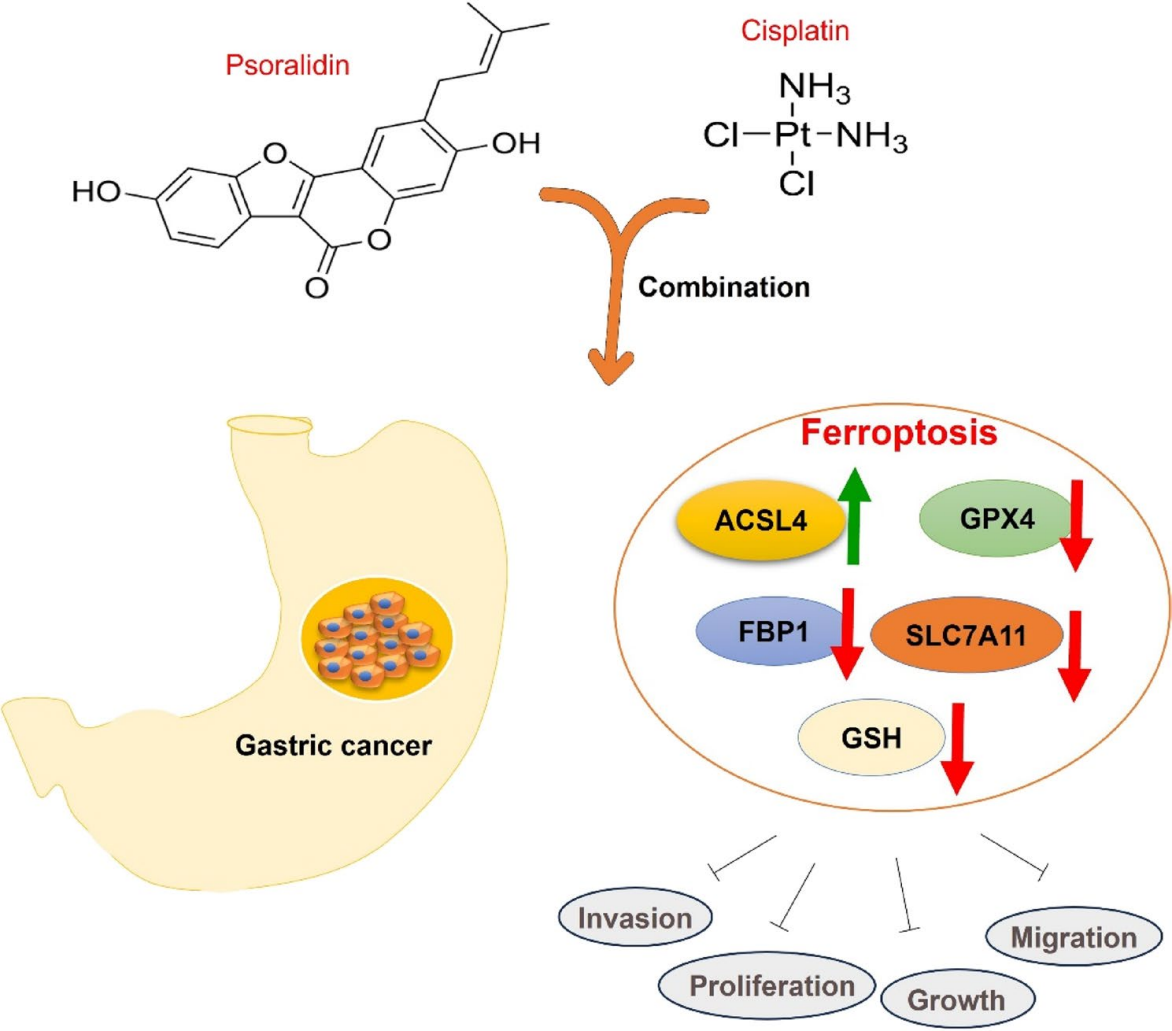

**Supplementary Information:**

The online version contains supplementary material available at 10.1186/s41065-025-00591-5.

## Introduction

Globally, gastric cancer (GC) ranks as the fifth most prevalent cancer and the fifth leading cause of cancer-related fatalities. In 2022, there were about 968,350 new cases and 659,853 new deaths of stomach cancer worldwide [1–3]. Surgery, radiation therapy, chemotherapy, and other therapies, such as hormone therapy and immunotherapy, have been regarded as the main strategies against cancers [4, 5]. GC is currently treated mainly with preoperative adjuvant chemotherapy and surgery combined with neoadjuvant chemotherapeutic agents (especially for late-stage GC cases) [6]. In China, the 1-, 2-, 3-, 4-, and 5-year survival rate for GC patients was about 74%, 55%, 44%, 38%, and 34%, respectively [7, 8]. Cisplatin (DDP) is a potent platinum-based antitumor agent. The combination of cisplatin with S-1 is recommended as a first-line chemotherapy against GC [9, 10]. The clinical applications of DDP still face challenges due to chemical resistance and side effects, including gastrointestinal reactions, kidney damage, peripheral neuropathy, myocardial damage, and bone marrow suppression [11, 12]. Alterations of programmed cell death forms, such as ferroptosis [13, 14], are involved in DDP resistance in GC. The combination of platinum-based antitumor agent, S-1 and immune checkpoint inhibitors can achieve promising efficiency against locally advanced GC. No significant postoperative complications were observed [15]. Thus, novel pharmacotherapies that can enhance antitumor effects and mitigate the toxic effects of chemotherapeutic drugs are needed for the treatment of GC.

*Psoralea corylifolia L.* is a medicinal herb with diverse ethnopharmacological and medicinal applications traditionally used for treating heart failure, cancer, psoriasis, and inflammation [16]. It contains several pharmacologically active compounds that have prominent antitumor functions [17–19]. For example, isobavachalcone, a natural chalcone compound isolated from *Psoralea corylifolia L.*, can suppress the proliferation and cause cell apoptosis of Panc02 cells. An in vivo assay revealed that isobavachalcone restrained tumor growth through activating antitumor immunity [17]. Psoralidin (PSO), an active compound found in the seeds of *Psoralea corylifolia L.*, has various biological effects, such as antioxidative, antitumor, and osteoprotective effects [20–22]. PSO causes apoptotic cell death in both human breast cancer cell lines (MCF-7) and human lung adenocarcinoma cell lines (A549), which suggests that PSO is a potential drug against cancer [23]. However, the role of PSO in GC remains elusive.

Herein, we explored the specific effects of PSO on the proliferation, migration, and invasion of GC cells. Considering the clinical challenges of chemotherapy in GC treatment, the combined antitumor effects of PSO and DDP were also investigated both in vitro and in vivo. The alterations of ferroptosis would also be determined. We also examined the organ damage of tumor-bearing nude mice following PSO/DDP administration to test the potential toxic effects. Through integrative bioinformatics analysis, we found ACSL4 as a potential target of PSO. Hence, we explored the dynamic interactions between PSO and ACSL4. Overall, this study aims to provide a potential strategy for the adjuvant therapy of GC.

## Materials and methods

### Cell culture and treatment

MKN-45 (Cat.No. CL-0292) and HGC-27 (CL-0107) cells (two GC cell lines) were purchased from Wuhan Pricella Biotechnology Co., Ltd. (Wuhan, China). Human HGC-27 cell line was isolated from the lymph node metastases of an undifferentiated gastric cancer patient, and MKN-45 cells were derived from liver metastases of a 62-year-old female patient with poorly differentiated gastric adenocarcinoma in Japan. Both types of cells have strong migration ability. The two cells with well growth status, which were passaged 3–6 times, were selected and trypsinized with trypsin (HyClone) at a concentration of 0.25%. The suspension was inoculated into 96-well plates uniformly at 1.0 × 10^4^ per well. Afterward, the cells were further cultured with RPMI-1640 (Gibco) supplemented with 10% fetal bovine serum (FBS) (Invitrogen, NY, USA). PSO (Cat.No. HY-N0232), the ferroptosis inhibitor ferrostatin-1 (Fer-1) (Cat.No. HY-100579), and the ferroptosis inducer Erastin (Cat.No.HY-15763) were obtained from MedChemExpress (New Jersey, USA). The GC cells were treated with varying doses of PSO (0–160 µM) [20, 21], Fer-1 (0.5 µM) [24], or Erastin (10 µM) [25].

### Cell counting kit-8 assay

MKN-45 and HGC-27 cells were seeded in plates with 96 cells (1 × 10^3^ cells/well) and cultured for 24 h. Next, PSO (0 to 160 µM) was added for treatment. One day later, CCK-8 solution (10 µL) was added to each well. The cells were maintained for 60 min in a 37 °C incubator. The absorbance was determined with a Bio-Rad spectrophotometer (CA, USA). The detection wavelength was 450 nm. A CCK-8 detection kit (Cat.No. CK04) was purchased from Dojindo Molecular Technologies (Kumamoto, Japan). CompuSyn software was used to compute the combination index (CI) of the drug combination effect.

### Colony formation assay

MKN-45 and HGC-27 GC cells in the well growth state were collected and subsequently seeded in a 6-well plate. A total of 1000 cells from each group were seeded in one well. After 7 days of culture to form colonies, PSO and/or DDP were administered to the cells. The cells were fixed with 4% paraformaldehyde following another 5-day incubation period. Each sample of fixed cells was stained with crystal violet solution. After the dye was removed, the number of colonies formed was counted.

### Edu assay

GC cells (MKN-45 and HGC-27) were seeded in 24-well plates, with each well containing 1*10^5^ cells. Twenty-four hours later, the cells were exposed to PSO, Fer-1, and/or DDP for 24 h. EdU solution (Cat. No. 40278ES25, Yeason, Shanghai, China), with a final concentration of 10 µM, was added to each well. Two hours later, after being washed twice with PBS, 4% paraformaldehyde was used for cell fixation. The cells were penetrated with 0.5% Triton X-100 solution. Next, 0.2 mL of Click iT reaction mixture was added to each well, and the mixture was incubated for 30 min. Then, 0.2 mL of DAPI solution was used for nuclear staining. Finally, a fluorescence microscope (Olympus, Tokyo, Japan) was used to observe the EdU-positive cells.

### Wound healing assay

The cells were seeded in 6-well plates at a concentration of 5 × 10^5^ per well. Once the cell monolayer reached 80–90% confluence, a wound was created in the cells via a pipette tip. PBS was used to wash the cells to remove cellular debris. The cells were subsequently cultured in serum-free medium. Cell proliferation was blocked with mitomycin C (10 µg/ml). The cells were incubated with 5 µM PSO, or/and 3 µM DDP. After 24 h, the images of the wounds were taken and the migrating rate of MKN-45 and HGC-27 cells was calculated.

### Transwell assay

Transwell assays were conducted to test the migration and invasion of MKN-45 and HGC-27 cells. The transwell chambers (Cat. No. 3422, 8 μm, Corning, NY, USA) were precoated with BD Matrigel (Cat. No.356234, BD, USA) in the cell invasion assay but not with BD Matrigel in the cell migration assay. GC cells (5 × 10^4^) in a volume of 200 µl of serum-free medium were seeded into the upper chambers. In the lower chamber, 600 µl of medium (containing 20% FBS) was added. The plates were placed in the incubator for 24 h. After fixation (4% paraformaldehyde), staining (0.1% crystal violet), and drying, the stained cells were photographed via an optical microscope (CX31, Olympus, Japan) and counted via ImageJ software.

### GSH, MDA, and LDH assays

The MKN-45 and HGC-27 GC cells were seeded in 24-well plates (1 × 10^5^ cells in each well). PSO, Fer-1, and/or DDP were used for a 24-hour treatment. Then, the cellular GSH, MDA and LDH levels were assayed via a GSH Assay Kit (Cat.No. S0053), an MDA Assay Kit (Cat.No. S0131S), and LDH Assay Kit (Cat.No. C0018S, Beyotime Biotechnology Co., Ltd., Shanghai, China).

### Analysis of Fe^2+^ levels

MKN-45 and HGC-27 cells were seeded in 24-well plates (1 × 10^5^ cells in each well). PSO, Fer-1, and/or DDP were used for a 24-hour treatment. An Fe^2+^ Assay Kit (E-BC-F101), which was obtained from Elabscience (China), was used to analyze the Fe^2+^ levels in the cell lysates. The detection procedures were conducted according to the manufacturer’s instructions. The optical density was measured at 593 nm via a microplate reader. Moreover, FerroOrange (F374, Dojindo, Kumamoto, Japan) was used to detect intracellular Fe^2+^ according to a previous study [26].

### Western blot (WB)

Following cell treatment, the GC cells were lysed with RIPA protein lysis buffer (Roche). The total protein was extracted via centrifugation. Next, 20 µg of total protein was loaded on a 12% polyacrylamide gel and subjected to two hours of electrophoresis at 100 V. The proteins were then transferred to PVDF membranes (Millipore, Bedford, MA, USA). The PVDF membranes were blocked with 5% skim milk at room temperature (RT) for one hour. The cells were incubated with antibodies against GPX4 (Cat No. 30388-1-AP, Proteintech, 1:1500), AIFM2 (Cat No. 20886-1-AP, Proteintech, 1:1500), SLC7A11 (ab307601, Abcam, 1:2000), ACSL4 (ab155282, Abcam, 1:1000), CBS (ab313382, Abcam, 1:1000), and β-actin (ab8226, Abcam, 1:3000) overnight at 4 °C. Horseradish peroxidase (HRP)-labeled anti-rabbit secondary antibody (1:3000) or anti-mouse antibody (1:3000) was used for incubation with the membranes after they were washed with TBST. Next, the membranes were subjected to three washes of 10 min each. Finally, a WB reagent (Invitrogen) was used for coloration, and each protein’s gray intensity was determined via ImageJ.

### Molecular docking analysis

Molecular docking analysis was conducted to evaluate whether PSO could bind directly to ACSL4, CBS, and GPX4. The structures of the CBS (7qgt) and GPX4 (6hn3) proteins were downloaded from the Protein Data Bank (PDB, http://www.rcsb.org/). The structure of ACSL4 (AF-O60488-F1) was downloaded from Alphafold (https://alphafold.ebi.ac.uk/). The structure of PSO (CID:5281806) was obtained from PubChem (https://pubchem.ncbi.nlm.nih.gov/). The compounds were processed via the LigPrep module in Schrödinger software (LigPrep, Schrödinger, LLC, New York, NY). The Epik method was used for protonation, hydrogenation, and energy optimization to output a 3D structure for docking at pH 7.0 ± 2.0. The docking of PSO against the above three proteins was simulated via AutoDock tools (v1.5.6). The alignment between target objects was measured via the PyMOL 2.3.0 system, and the binding energy was used to predict the possibility of direct binding.

### Cellular thermal shift assay (CETSA)

CETSA was carried out to determine the binding between PSO and ACSL4. The GC cells (MKN-45 and HGC-27) were pretreated with PSO (40 µM) or DMSO, and heated at different temperatures, including 45, 50, 55, 60, 65, and 70 °C. After a 4-min incubation, the cells were put in a temperature at 4 °C and lysed. Total protein was extracted by centrifugation at 14,000 × g. Western blot was used to detect ACSL4, GPX4, and SLC7A11 in the total protein.

### Xenograft experiments in nude mice

Twenty-four BALB/c nude mice (6 weeks old, 19 g ± 2 g), which were purchased from the Experimental Animal Center of Nanchang University, were reared under specific pathogen-free (SPF) conditions. MKN-45 cells at the logarithmic growth stage were trypsinized and made into single-cell suspensions (2 × 10^7^ cells/mL). Then, 0.1 mL of the suspension was subcutaneously injected into the right axilla of each nude mouse. The mice were reared under SPF conditions, and their diet, activity and general condition were monitored. When the tumor volume reached 50 mm^3^, the mice were randomized into four groups, which were administered saline or PSO (15 mg/kg) via oral gavage once every two days [21] or DDP (3 mg/kg, i.p., once every 2 days) [27]. The tumor size and weight were measured every 3 days and their survival was checked before meeting criteria for euthanasia. On the 28th day or when tumors reached 1.5 cm in length or when the body weight of mice have reduced by 20%, the mice were anesthetized by CO_2_ inhalation for 5 min. The mice were sacrificed by cervical dislocation. In the euthanasia process, the mice stayed in cages with a clean and comfortable environment. Enough food and water were provided, and the temperature and humidity were maintained at the right conditions to minimize the mice’s anxiety. The tumors were removed, and their volume and weight were measured. All animal experiments were approved by the ethics committee of The First Hospital of Nanchang (Approval No.2022011-007). The experiments were conducted by well-trained laboratory technicians.

### H&E staining and immunohistochemistry (IHC)

Mouse tumor, and five organ tissues were fixed in 4% paraformaldehyde and paraffin-embedded. The sections were subsequently dewaxed, hydrated, and subjected to antigen retrieval. An H&E staining kit was used to detect histopathological changes of organs and tumor tissues. The sections were incubated with immunostaining blocking solution for 1 h, followed by incubation with anti-Ki-67 (ab16667, Abcam, 1:200) or anti-TUNEL (ab206386), Abcam, 1:200) antibodies overnight at 4 °C. Next, they were rinsed with PBS and incubated for one hour with biotinylated goat anti-rabbit IgG (1:500; ab207995, Abcam) at RT. Subsequently, the sections (incubated with anti-Ki-67) were subjected to rewashing and coloring for 1 min with 3,3’-diaminobenzidine hydrochloride. Finally, the sections were rinsed with PBST, dyed with hematoxylin II for one minute, and reviewed with a light microscope at a magnification of 200× (CX31, Olympus, Japan).

### Serum AST, CRE, BUN and AST analysis

The levels of liver enzymes (ALT and AST) and kidney function markers (BUN and Cre) in the serum were determined via appropriate detection kits following the manufacturers’ instructions. Commercial kits, including alanine aminotransferase (ALT) (Cat.No. E-BC-K235-S), AST (aspartate aminotransferase) (Cat.No. E-BC-K236-M), BUN (urea) (Cat.No. E-BC-K183-M), Cre (creatinine) (Cat.No. E-BC-K188-M) were all purchased from Elabscience (China).

### Identification of ferroptosis-associated targets in GC

We analyzed the differentially expressed (GE) genes in stomach adenocarcinoma (STAD) via GEPIA2 (http://gepia2.cancer-pku.cn/#index), an online tool based on large TCGA and GTEx datasets. Genes whose fold change (FC) was greater than 1 and whose p value was < 0.01 were selected. The pathologic genes associated with STAD were subsequently analyzed via LinkedOmics (https://www.linkedomics.org/login.php) with *P* < 0.01 as the selection criterion. In addition, GeneCards (https://www.genecards.org/) was used to identify ferroptosis-associated genes. Only protein-coding genes with scores no less than 2 were selected.

### Statistical analysis

SPSS 21.0 software (SPSS Inc., Chicago, IL, USA) was used for data analysis, and the results are presented as the means ± SDs (x ± s). The data were analyzed by either one- or two-way analysis of variance followed by Dunnett’s and Bonferroni post hoc correction. *P* < 0.05 indicated statistical significance.

## Results

### Synergistic effects of the combination of PSO and DDP in repressing GC cell proliferation

**Fig. 1:**
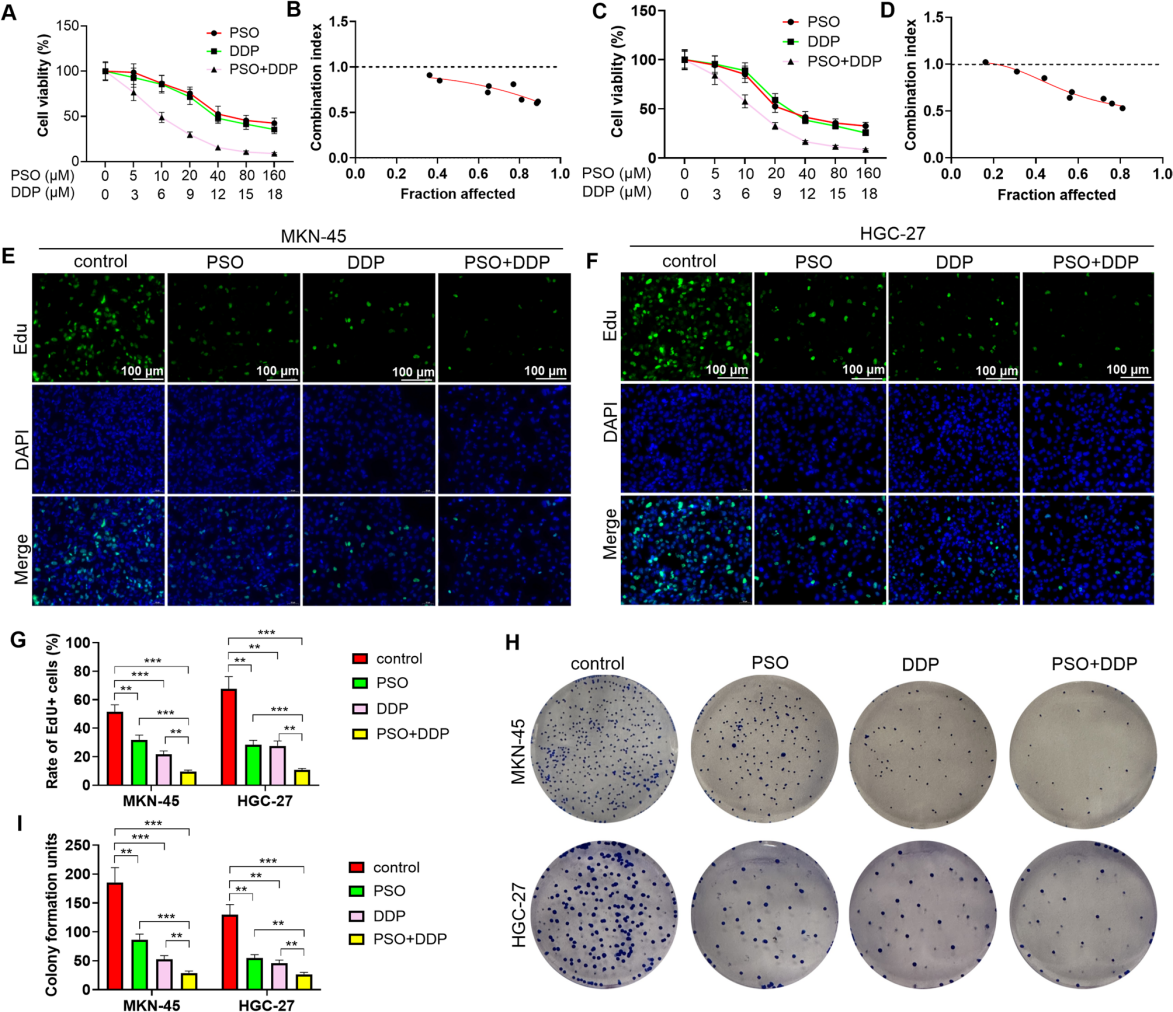
PSO and DDP hindered GC cell proliferation. GC cells (MKN-45 and HGC-27) were treated with PSO (0 to 160 µM) with or without DDP (0–18 µM). **A** and **C**. Cell viability was monitored by a CCK-8 assay. **B** and **D**. Compusyn software was used to calculate the combination index (CI). **E**-**G**. An EdU staining assay was conducted to evaluate GC cell proliferation. Scale bar = 100 μm. **H**-**I**. A colony formation assay was performed with MIN-45 and HGC-27 cells treated with PSO or/and DDP. ** *P* < 0.01, *** *P* < 0.001. *N* = 3

To determine the antitumor effects of PSO in GC, we treated GC cells (MKN-45 and HGC-27) with various concentrations of PSO (0 to 160 µM) with or without DDP. The results of the CCK-8 assay revealed that treatment with PSO or DDP alone led to a reduction in GC cell viability. The combination of PSO and DDP further inhibited cell viability (Fig. 1A and C). The combined index (CI) analysis revealed that when the inhibitive rate was over 0.81 (for MKN-45 cells) or over 0.56 (for HGC-27 cells), the CI was less than 0.7, suggesting that PSO and DDP had synergistic effects on the proliferation of the two GC cell lines (*P* < 0.05, Fig. 1A-D). Then, the GC cells (MKN-45 and HGC-27) were exposed to PSO (20 µM) and/or DDP (9 µM) for 24 h. EdU staining and colony formation assays revealed that PSO and DDP dramatically impeded GC cell proliferation (*P* < 0.05, Fig. 1E-H). The combination of PSO and DDP had prominent suppressive effects on GC cell proliferation (*P* < 0.05, Fig. 1E-H). In conclusion, PSO and DDP synergistically suppressed GC cell proliferation in vitro.

### Effects of the combination of PSO and DDP on GC cell migration and invasion

**Fig. 2:**
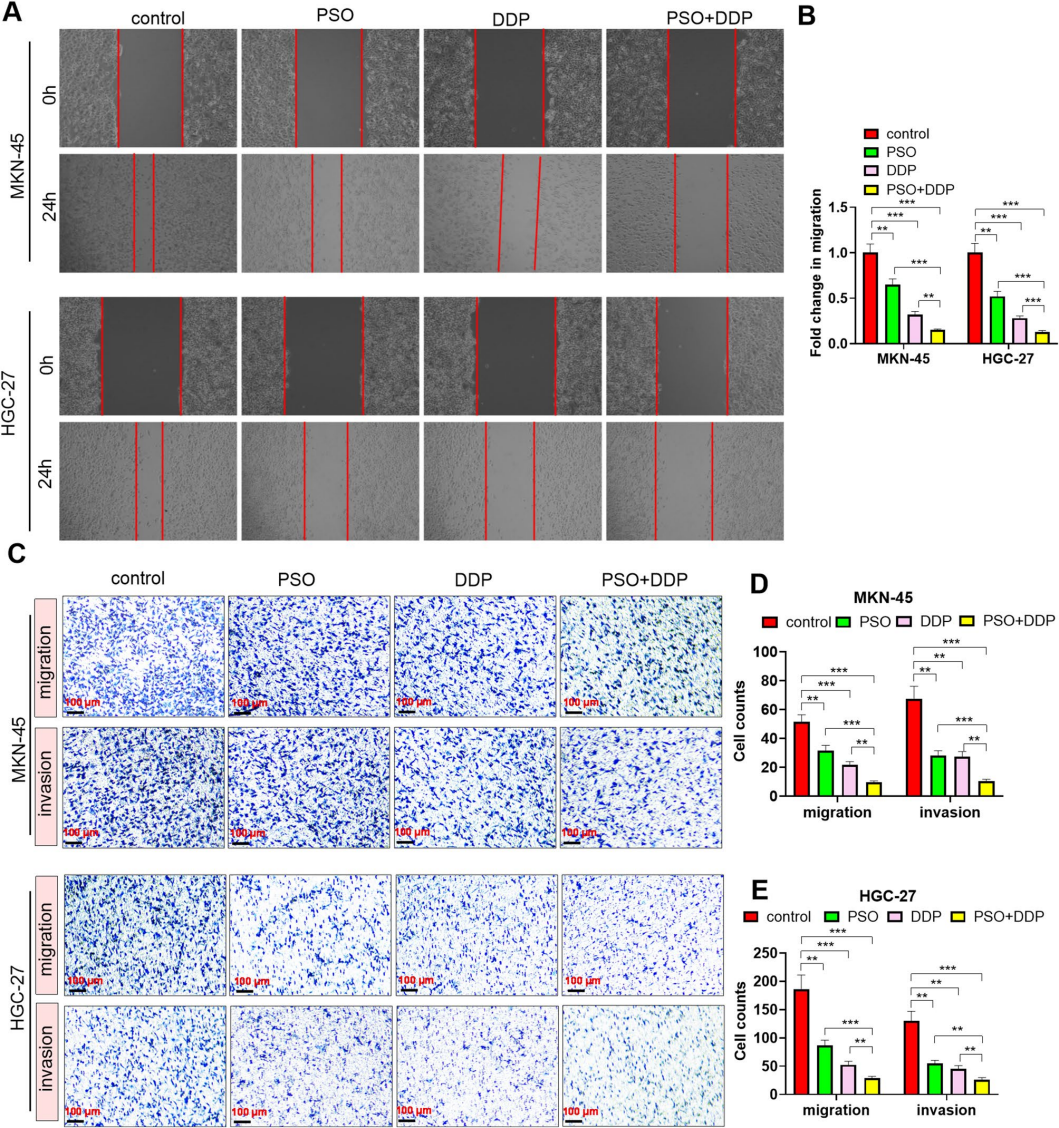
The combination of PSO and DDP affected the migration and invasion of GC cells. GC cells (MKN-45 and HGC-27) were treated with PSO (5 µM) and/or DDP (3 µM).** A**-**B**. Cell migration was determined via a wound healing assay. **C**‒**D**. Cell migration and invasion were measured via a transwell assay. Scale bar = 100 μm. ** *P* < 0.01, *** *P* < 0.001. *N* = 3

We treated GC cells (MKN-45 and HGC-27) with different concentrations of PSO (5 µM) and/or DDP (3 µM) to clarify the impact of PSO on the migration and invasion of GC cells. Wound healing and transwell assays demonstrated that PSO and DDP reduced the migrative and invasive functions of GC cells (Fig. 2A-E, *P* < 0.05). Moreover, the combination of PSO and DDP significantly suppressed GC cell migration and invasion (Fig. 2A-E, *P* < 0.05 vs. the PSO or DDP group).

### The combination of PSO and DDP repressed GC cell growth in vivo

**Fig. 3:**
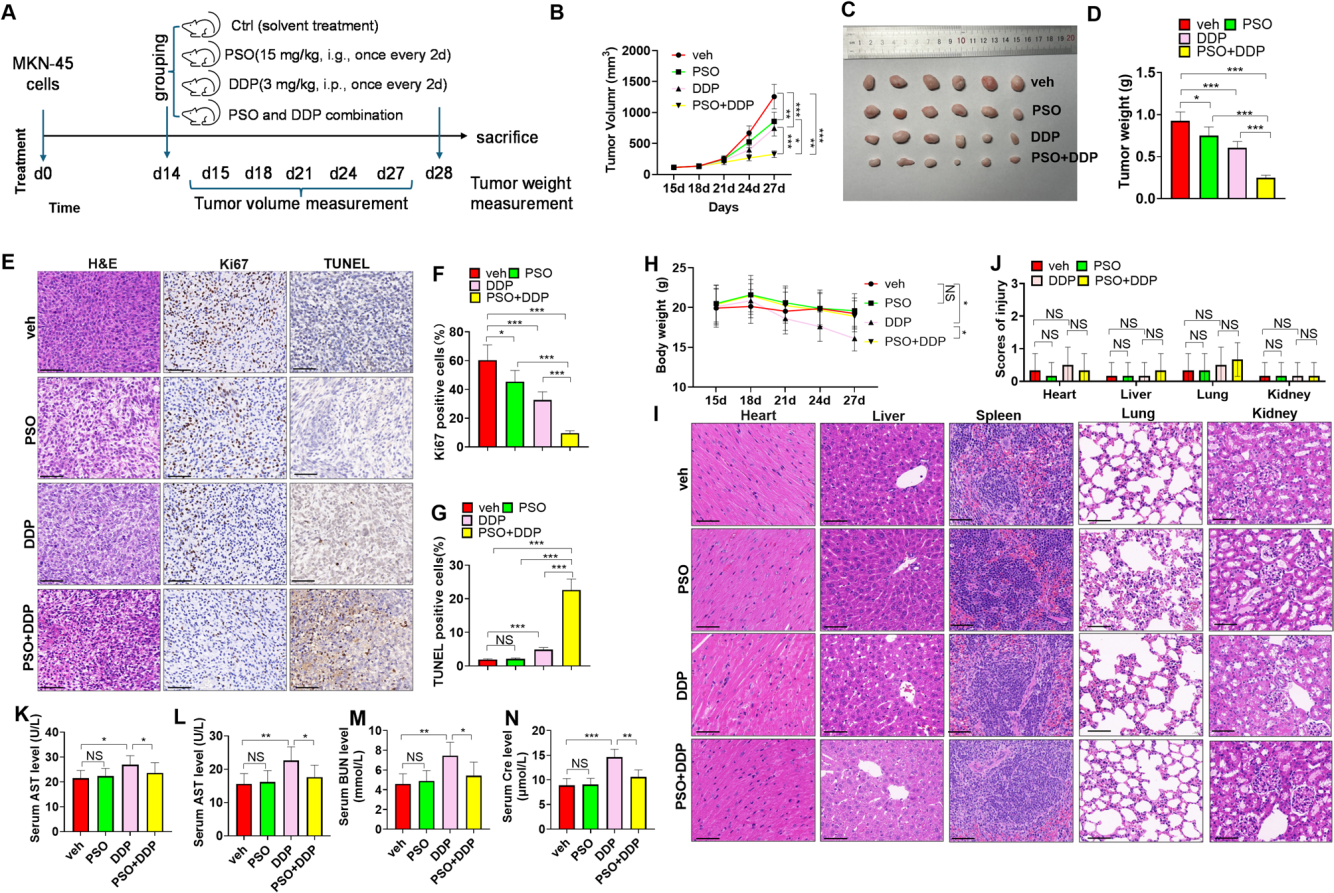
PSO and DDP attenuated GC cell growth in vivo. GC cells (MKN-45) were injected subcutaneously into BALB/c nude mice to establish xenograft models. The mice were treated with PSO or/and DDP (**A**). **B**-**D**: Tumor volume, images and weight of the mice. **E**: **H**&**E** staining, Ki-67 staining, and TUNEL staining were conducted. **F**. The percentage of Ki67-positive cells was determined. **G**. Cell apoptosis was evaluated by the percentage of TUNEL-positive cells. staining assay. **H**. The body weights of the nude mice during the experimental procedures. **I**-**J**. H&E staining was performed to assess the histological morphology of the main organs, including the heart, liver, spleen, lung, and kidney, of the mice. The scores of organ injuries were counted. **K**‒**N**. Liver and kidney functions were evaluated by examining the serum levels of AST (**J**), ALT (**K**), BUN (**L**), and Cre (**M**). Scale bar = 100 μm. NS *P* > 0.05, * *P* < 0.05, ** *P* < 0.01, *** *P* < 0.001. *N* = 6

A xenograft model in nude mice was constructed, and the mice were treated with PSO, DDP, or their combination (Fig. 3A). We measured the tumor volume and weight. In contrast with the veh group, the sharpest decreases in tumor volume and weight were observed after PSO or DDP treatment (*P* < 0.05, Fig. 3B-D). Compared with single treatment with PSO or DDP, the combination of PSO and DDP had more prominent effects on inhibiting tumor cell growth (*P* < 0.05, Fig. 3B-D). The IHC results revealed a decrease in the Ki67-positive cell percentage after PSO + DDP treatment (*P* < 0.05 vs. the PSO or DDP group; Fig. 3E-F), whereas the percentage of TUNEL-positive cells was significantly greater (*P* < 0.05 vs. the PSO or DDP group; Fig. 3E, G). To test the toxic effects of PSO and DDP on the nude mice, their body weights were measured. Longer PSO treatment did not reduce body weight, whereas DDP reduced body weight. However, the combination of PSO and DDP markedly increased body weight (*P* < 0.05 vs. DDP group; Fig. 3H). The histological morphology of the main organs, including the heart, liver, spleen, lung, and kidney, of the mice was further examined via H&E staining. No significant differences in organ injury scores among the four groups were found (Fig. 3I-J). By evaluating liver and kidney functions via examination of the serum levels of ALT, AST, BUN, and Cre, we discovered that DDP treatment, but not PSO treatment, significantly increased the serum ALT, AST, BUN, and Cre levels. Compared with the DDP group, the PSO and DDP combination treatment obviously improved the DDP-mediated toxic effects (Fig. 3K-N).

### The combination of PSO and DDP induced ferroptosis in GC cells

**Fig. 4:**
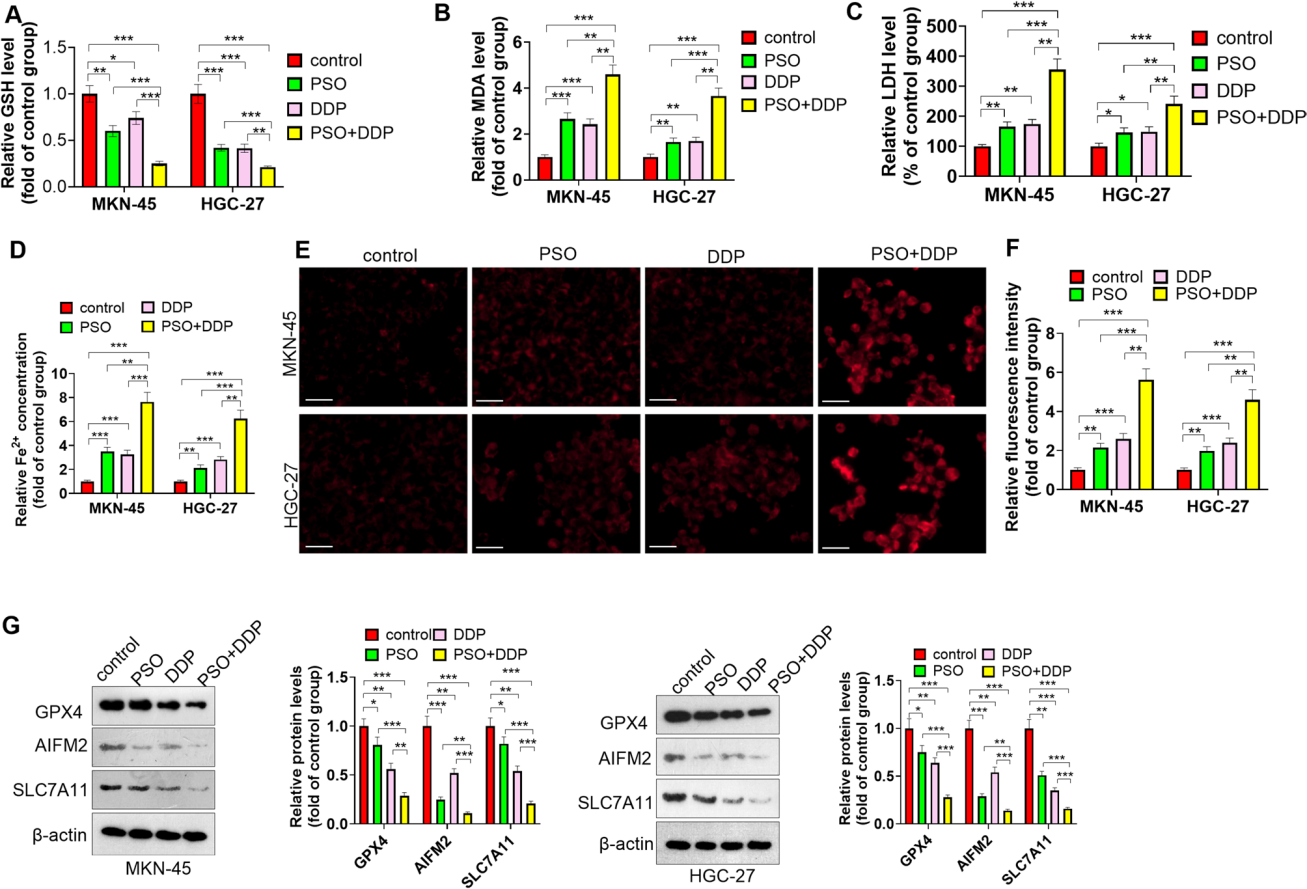
Effects of the combination of PSO and DDP on ferroptosis in GC cells. **A**-**C**. GSH, MDA and LDH production in two GC cell lines (MKN-45 and HGC-28) was measured via commercial detection kits. **D**-**E**. Fe^2+^ levels in both MKN-45 and HGC-27 cells were evaluated via an Fe^2+^ assay kit (**D**) and FerroOrange staining (**E**-**F**). **G**. Western blotting was conducted to test the protein levels of GPX4, AIFM2, and SLC7A11 in GC cells. Scale bar = 100 μm. * *P* < 0.05, ** *P* < 0.01, *** *P* < 0.001. *N* = 3

To further investigate the mechanisms of PSO in GC cells, we measured GSH, MDA and LDH production in two GC cell lines (MKN-45 and HGC-28). Single PSO or DDP treatment reduced GSH levels and increased MDA and LDH levels (Fig. 4A-C). The combination of PSO and DDP further suppressed GSH levels and promoted MDA and LDH levels (vs. the PSO or DDP group, Fig. 4A-C). By measuring Fe^2+^ levels, we found that single PSO or DDP administration increased Fe^2+^ levels in both MKN-45 and HGC-27 cells, and this increase was further induced by the combination of PSO and DDP (Fig. 4D-F). We conducted western blotting to test the protein levels of GPX4, AIFM2, and SLC7A11, three vital ferroptosis-related proteins. We found that PSO and DDP treatment reduced GPX4, AIFM2, and SLC7A11 expressions. Interestingly, the combination of PSO and DDP further inhibited GPX4, AIFM2, and SLC7A11 expressions (vs. the PSO or DDP group, Fig. 4G). Thus, PSO and DDP cause ferroptosis in GC cells.

### Inhibiting ferroptosis partially reversed the antitumor effects of PSO and DDP in GC

**Fig. 5:**
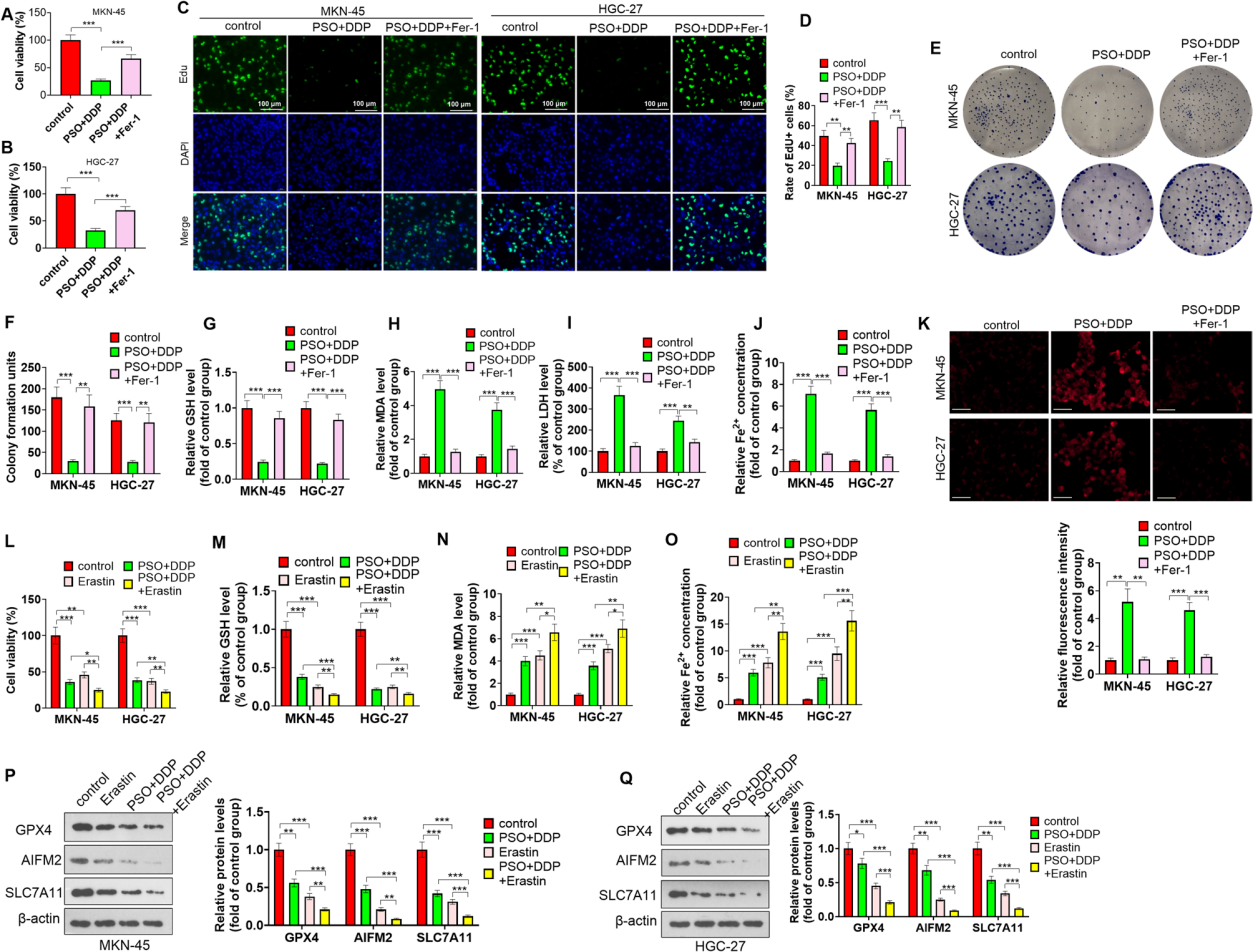
Effects of ferroptosis inhibition on PSO- and DDP-mediated antitumor effects. GC cells (MKN-45 and HGC-27) were treated with PSO and DDP and/or the ferroptosis inhibitor Fer-1. **A**-**B**. **A** CCK8 assay was conducted to test cell viability. **C**-**F**. Proliferation was tested by EdU staining (**C**-**D**) and colony formation (**E**-**F**). **G**-**I**. GSH, MDA and LDH production in two GC cell lines (MKN-45 and HGC-28) was tested. **J**‒**K**. Fe^2+^ levels were tested via an Fe^2+^ assay kit (**J**) and FerroOrange staining (**K**). GC cells (MKN-45 and HGC-27) were treated with PSO and DDP and/or the ferroptosis inducer Erastin. L. A CCK8 assay was conducted to test cell viability. **M**-**N**. GSH and MDA levels in two GC cell lines (MKN-45 and HGC-28) were tested. O. Fe^2+^ levels were tested via an Fe^2+^ assay kit. P-Q. Western blotting was conducted to test the protein levels of GPX4, AIFM2, and SLC7A11 in GC cells. Scale bar = 100 μm. * *P* < 0.05, ** *P* < 0.01, *** *P* < 0.001. *N* = 3

To confirm the specific roles of ferroptosis in PSO**-** and DDP-mediated antitumor effects, we treated GC cells (MKN-45 and HGC-27) with the ferroptosis inhibitor Fer-1. Compared with the PSO + DDP group, Fer-1 addition increased cell viability (Fig. 5A-B). Cell proliferation was tested by EdU staining and colony formation assay, and the results showed that Fer-1 treatment enhanced the proliferative ability of MKN-45 and HGC-27 cells (Fig. 5C-F). The GSH, MDA, and LDH production in the two GC cell lines (MKN-45 and HGC-28) was tested. Fer-1 treatment increased GSH levels and reduced MDA and LDH levels (Fig. 5G-I). By measuring Fe^2+^ levels, we found that, compared with the PSO + DDP combination, Fer-1 treatment reduced Fe^2+^ levels (Fig. 5J-K). Thus, Fer-1 inhibited PSO**-** and DDP-induced ferroptosis in GC cells.

To rule out possible off-targets on ferroptosis, Erastin, a ferroptosis inducer, was treated with MKN-45 and HGC-27 cells without or with PSO. It was found that Erastin treatment reduced cell viability and combining Erastin and PSO further repressed cell viability (Fig. 5L). The GSH level was reduced following Erastin treatment, while MDA and Fe2 + levels were increased by Erastin. Co-administration of Erastin and PSO further reduced GSH level and elevated MDA and Fe2 + levels (Fig. 5M-O). Western blot results indicated that Erastin inhibited GPX4, AIFM2, and SLC7A11 protein levels. Erastin and PSO combination can further restrain their profiles (Fig. 5P-Q).

### PSO reversed GC malignancies by targeting ACSL4

**Fig. 6:**
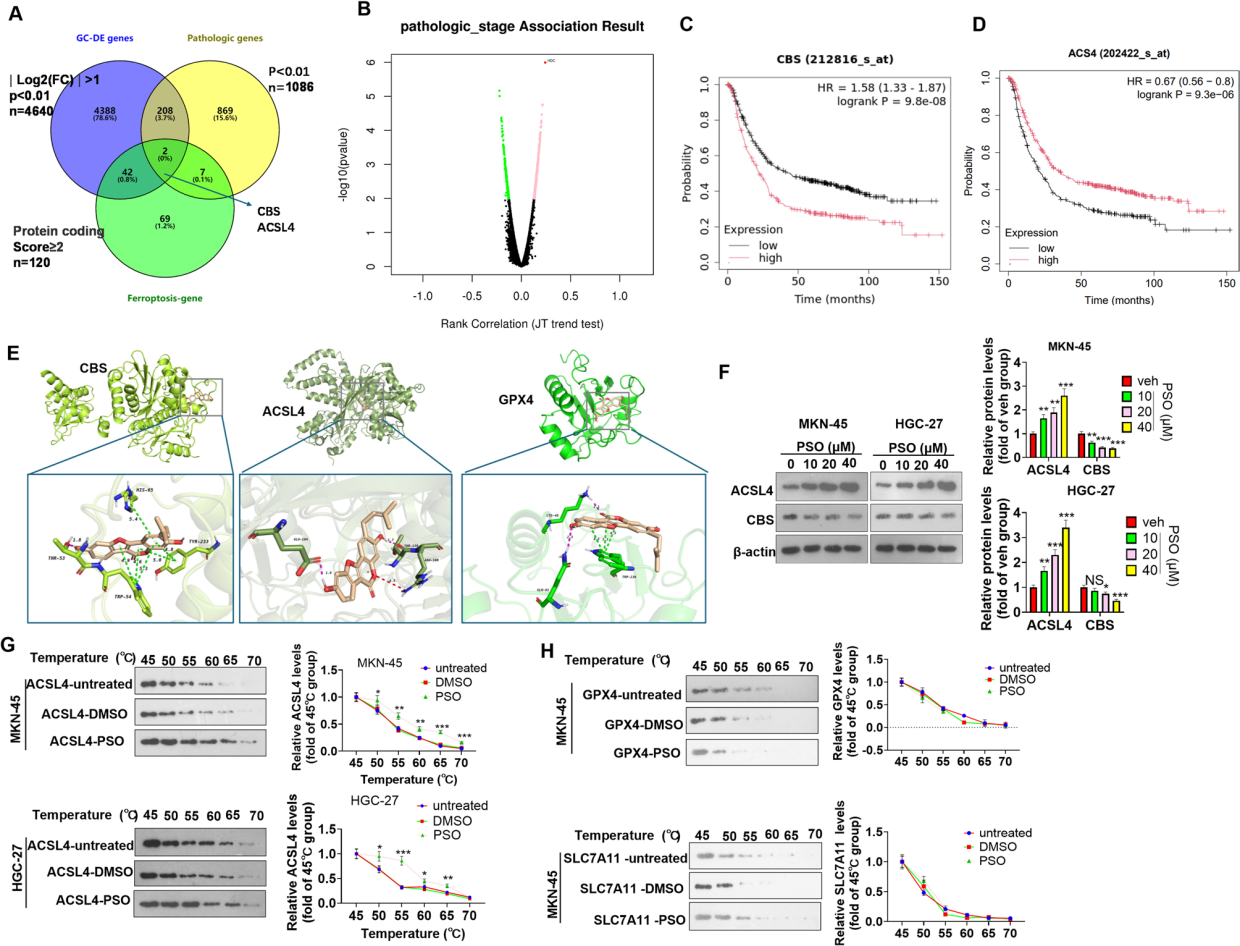
Potential targets of the PSO in GC.** A** Venn diagram analysis was conducted to identify GC-DE genes, pathologic-associated genes, and ferroptosis-associated genes. Two genes, CBS and ACSL4, were identified. **B** Volcano plot of patients’ stage-associated genes in STAD. **C**‒**D**. Kaplan‒Meier plotter images of the associations of different levels of CBS and ACSL4 with the overall survival of patients with STAD. **E**. Visualization of the molecular docking of PSO with CBS (binding energy: −5.143 kcal/mol), ACSL4 (binding energy: −5.889 kcal/mol), and GPX4 (binding energy: −4.092 kcal/mol). **F**. Western blotting was conducted to detect ACSL4 and CBS levels in MKN-45 and HGC-27 cells treated with PSO (10–40 µM). NS *P* > 0.05, * *P* < 0.05, ** *P* < 0.01, *** *P* < 0.001 vs. the veh group. *N* = 3. **G**-**H**. CETSA was conducted to test the binding of PSO and ferroptosis-associated proteins. ACSL4, GPX4 and SLC7A11 levels were detected via western blotting. * *P* < 0.05, ** *P* < 0.01, *** *P* < 0.001 vs. the DMSO group. *N* = 3

**Fig. 7:**
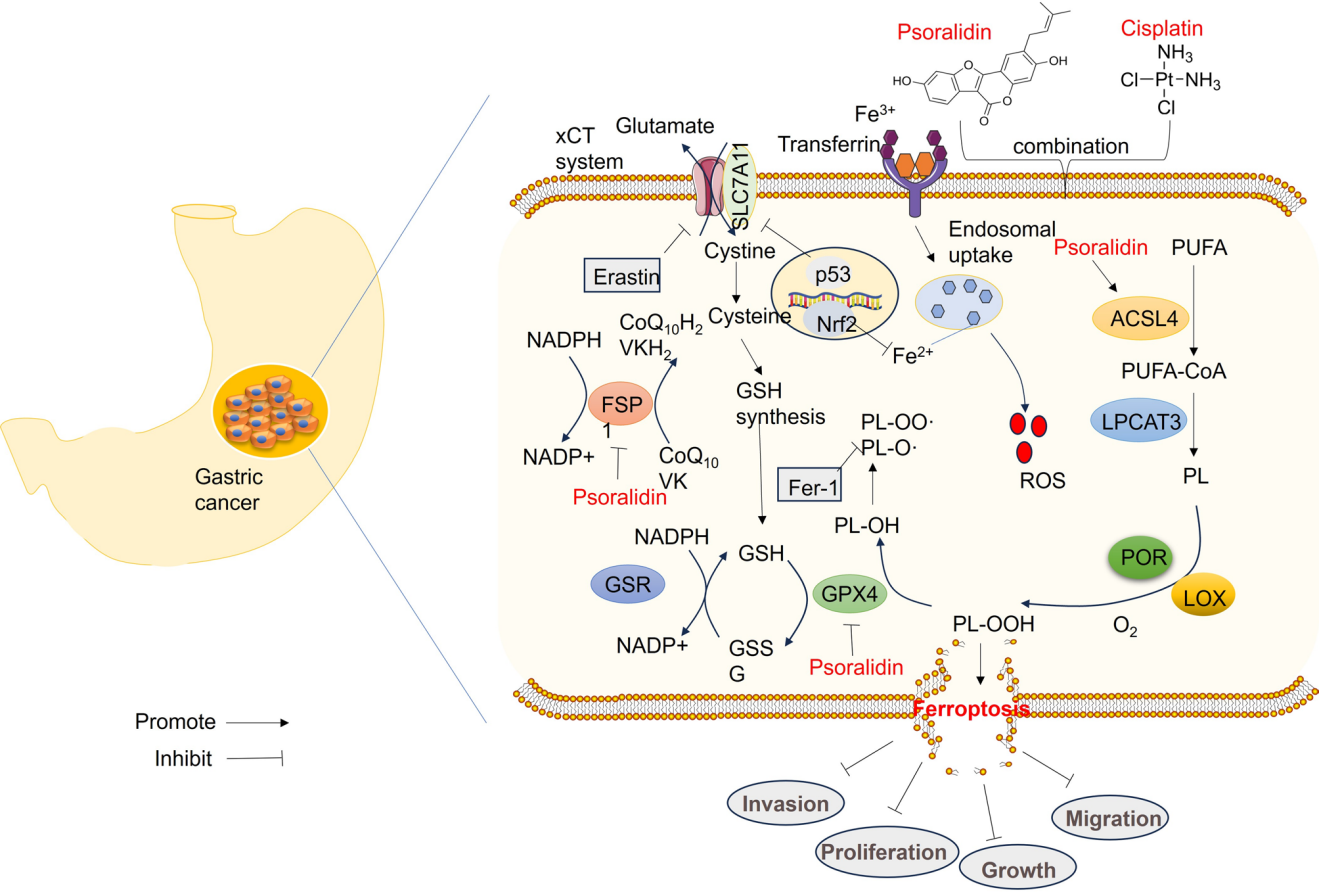
A sketch of the mechanism. The combination of PSO and DDP has antitumor effects on GC by repressing proliferation, migration, and invasion via the induction of ferroptosis via the promotion of ACSL4 and the inhibition of GPX4

To further investigate the specific targets of PSO in GC cells, we analyzed ferroptosis-associated genes in GC and found that CBS and ACSL4 were included via Venn diagram analysis (Fig. 6A). A volcano plot of patients’ stage-associated genes in STAD is shown in Fig. 6B. The Kaplan‒Meier plotter (https://kmplot.com/analysis/) was used to analyze the associations of CBS and ACSL4 with the overall survival of patients with STAD. Higher CBS levels and lower ACSL4 levels were associated with poorer overall survival in patients with STAD (Fig. 6C-D). Molecular docking was conducted to visualize the binding of PSO with CBS (binding energy: −5.143 kcal/mol), ACSL4 (binding energy: −5.889 kcal/mol), and GPX4 (binding energy: −4.092 kcal/mol) (Fig. 6E). Western blot analysis revealed that PSO treatment significantly promoted ACSL4 and inhibited CBS levels in the two GC cell lines (Fig. 6F). The CETSA curves indicated that PSO increased the thermal stability of the ACSL4 protein in both MKN-45 and HGC-27 cells (Fig. 6G). However, the thermal stability of GPX4 and SLC7A11 was not significantly altered following PSO treatment (Fig. 6H). Hence, the role of the combination of PSO and DDP in suppressing GC cell malignancies was achieved via ACSL4-mediated ferroptosis (Fig. 7).

## Discussion

As recent reports have demonstrated, PSO exhibits antitumor activity in human cancers, such as liver cancer [28], colon cancer [29] and prostate cancer [30]. Through in vitro and in vivo experiments, we confirmed that PSO enhanced ferroptosis in GC cells and impeded tumor growth. Moreover, we found that the combination of PSO and DDP had synergistic antitumor effects on GC.

An increasing number of studies have shown that active compounds isolated from *Psoralea corylifolia* seeds, including bavachin [31], bakuchiol [32], psoralen and isopsoralen [33], can exhibit antitumor functions. These studies highlight the potential of antitumor compounds from *Psoralea corylifolia* seeds. As an important component of this herb, PSO can suppress cancer cell viability and enhance apoptosis in a direct way, which has been verified previously [21, 23, 28–30]. In addition, PSO induces elevated production of reactive oxygen species (ROS) and causes DNA damage by mediating caspase 3/7 activation and c-Jun N-terminal kinase 1/2 activation [34]. In addition, PSO markedly restrained cell proliferation and promoted autophagy and DNA damage in breast cancer cells [35]. Recently, PSO has shown prominent immunostimulatory activity during tumor progression [36]. In the present study, we found that PSO inhibited the proliferation, migration, and invasion of GC cells and that these antitumor functions were more significant when combined with DDP treatment. The in vivo assay also confirmed that the combination of PSO and DDP inhibited GC cell growth. Therefore, PSO may be a novel adjuvant therapy for GC.

Multiple side effects of DDP in GC treatment have been found [37]. Previous studies have found that DDP contributed to acute kidney injury [38] and liver injury [39] by inducing oxidative stress and apoptosis. PSO has protective effects against sepsis-induced acute lung injury [40] and myocardial injury [41] at a dose of 50 mg/kg. PSO also prevented sepsis-induced liver injury (indicated by reduced AST level) and renal injury (indicated by reduced BUN level). In terms of mechanisms, PSO restrained TLR2-mediated inflammatory NF-κB signaling and oxidative stress. In this study, we found that compared with the DDP group, PSO and DDP combination exhibited reduced renal and liver injuries, as indicated by serum levels of AST, ALT, BUN, and Creatinine (Fig. 3). We supposed that PSO might prevent DDP-associated toxic effects.

GC is characterized by uncontrolled cell growth and proliferation, which can be influenced by genetic alterations, including ferroptosis [42]. Ferroptosis is a form of regulated cell death characterized by iron-dependent lipid peroxidation and the accumulation of ROS, leading to membrane damage and cell death [43]. Xiao et al. investigated the correlations of ferroptosis-associated genes with the development of GC. The signature of ferroptosis-related genes is significantly correlated to the prognosis of patients with STAD [44]. Another study suggests that GPX4, AIFM2, and ACSL4 have higher levels in STAD, and AIFM2 expression level had a negative correlation with the infiltration of immune cells, including CD4^+^ T cells, CD8^+^ T cells, macrophages, neutrophil cells and dendritic cells [45]. Ferroptosis has been suggested to act as a tumor suppressor mechanism by inducing selective cell death in GC cells. GC cells frequently exhibit alterations in iron metabolism and lipid peroxidation pathways, increasing their susceptibility to ferroptosis. Triggering ferroptosis by promoting CST1 in GC cells could inhibit tumor growth and metastasis [46]. On the other hand, cancer cells can also develop mechanisms to evade ferroptosis and develop chemical resistance to this form of cell death [47]. For example, activating transcription factor 3 (ATF3) expression is reduced in GC cells and GC tissues, and lower ATF3 expression in cisplatin-resistant GC cells is observed. The GC cells with DDP resistance presented lower MDA, lipid peroxidation, and ROS levels but higher intracellular GSH levels. ATF3 overexpression induced ferroptosis in DDP-resistant GC cells by inhibiting the activation of Nrf2/Keap1/xCT (SLC7A11) signaling [48]. Here, we found that PSO can decrease GSH levels and increase MDA and LDH levels in GC cells. In addition, PSO increased Fe^2+^ levels but inhibited GPX4, AIFM2 (also known as FSP1), and SLC7A11 expression. Moreover, the combination of PSO and DDP had more significant effects on the above alterations. Hence, PSO potentially exerts synergistic antitumor effects with DDP in GC through the mediation of ferroptosis.

Acyl-CoA synthetase long-chain family member 4 (ACSL4) is an enzyme that plays a key role in lipid metabolism and is involved in the conversion of long-chain fatty acids to acyl-CoA esters [49]. ACSL4 preferentially catalyzes polyunsaturated fatty acids (PUFAs) into PUFA-CoA [50]. Activated PUFAs are then transferred to phospholipids by lysophosphatidylcholine acyltransferases (LPCATs), forming phospholipids with PUFA side chains (e.g., PE-AA, PE-LA). These phospholipids are embedded in cell membranes, where they become substrates for lipoxygenases (LOXs)—enzymes that catalyze their peroxidation into lipid peroxides (LPO) [51, 52]. When the GSH-GPX4 system (which reduces LPO to non-toxic alcohols) is compromised (e.g., via GPX4 inhibition or GSH depletion), LPO accumulates rapidly, leading to membrane damage and ferroptosis [53]. Genetic knockout or pharmacological inhibition of ACSL4 abolishes PUFA-CoA synthesis, reduces membrane PUFA-containing phospholipids, and renders cells resistant to ferroptosis inducers [54]. Emerging evidence suggests that ACSL4 may influence cancer development and progression by influencing tumor growth, metastasis, lipid metabolism, drug resistance, radioresistance, and cell death pathways [55–58]. For example, ACSL4 was elevated in radioresistant breast cancer cells. Downregulating ACSL4 potentiated tumor cells to irradiation and inhibited cell migration [58]. Following treatments with platinum-based drugs, melanoma cells exhibited higher formation of lipid rafts in the membrane mediated by the ACSL4-LPO axis. Removing membrane cholesterol can dislocate ACSL4-mediaged lipid rafts, leading to enhanced immunogenic cell death and higher antitumor effectiveness of platinum-based drugs [59]. Recently, ACSL4 was found to mediate metastatic extravasation of metastasis-derived tumor cells by enhancing membrane fluidity. Double repression of ACSL4 and enoyl-CoA hydratase 1 (ECH1) markedly reversed the metastasis of tumor cells [60].

Here, we found that ACSL4 is a ferroptosis-associated gene in GC and that higher ACSL4 levels can predict better overall survival in GC patients. A recent study revealed that curcumin has antitumor effects on GC by mediating cell viability, death, and ferroptosis via the suppression of GPX4 and SLC7A11 and the promotion of ACSL4 [61]. Tanshinone I (Tan I), a bioactive component of *Salvia miltiorrhiza*, has an antitumor effect on GC by increasing the MDA, ROS, and Fe^2+^ contents. In addition, Tan I represses GPX4, SLC7A11, and FTH1 while promoting TFR1 and ACSL4 [62]. Thus, these studies indicate that ACSL4 is a potential antitumor target in GC. Here, we found that PSO can directly bind ACSL4 and increase its protein level in GC cells, suggesting that PSO can inhibit GC development by targeting ACSL4.

However, several limitations remain in the present study. First of all, the antitumor effects of PSO were verified on two GC cell lines. Future investigations should be conducted on patient-derived xenografts (PDXs) or primary GC cells to validate translational relevance [63–67]. Second, our cellular assays confirmed that repressing ferroptosis reversed PSO-mediated antitumor effects, which require further animal studies for identification. Third, considering the synergistic effects of PSO and DDP against GC, the exploration of whether PSO can affect DDP resistance in GC would help the clinical translation of PSO in adjuvant therapy of GC.

Overall, our study revealed that PSO and DDP have synergistic antitumor effects by curbing GC cell growth, migration and invasion through the induction of ferroptosis via the upregulation of ACSL4 (Fig. 7). PSO may become a potential adjuvant therapy drug for treating GC.

## Supplementary Information

Supplementary Material 1.

## Data Availability

The datasets used and analyzed during the current study are available from the corresponding author upon reasonable request.
