# Supplementary figures and images for: The synergistic antitumor effects of psoralidin and cisplatin in gastric cancer by inducing ACSL4-mediated ferroptosis
Source: Hereditas. 2025 Nov 4;162:223. doi: 10.1186/s41065-025-00591-5 (PMC12584398; doi:10.1186/s41065-025-00591-5)

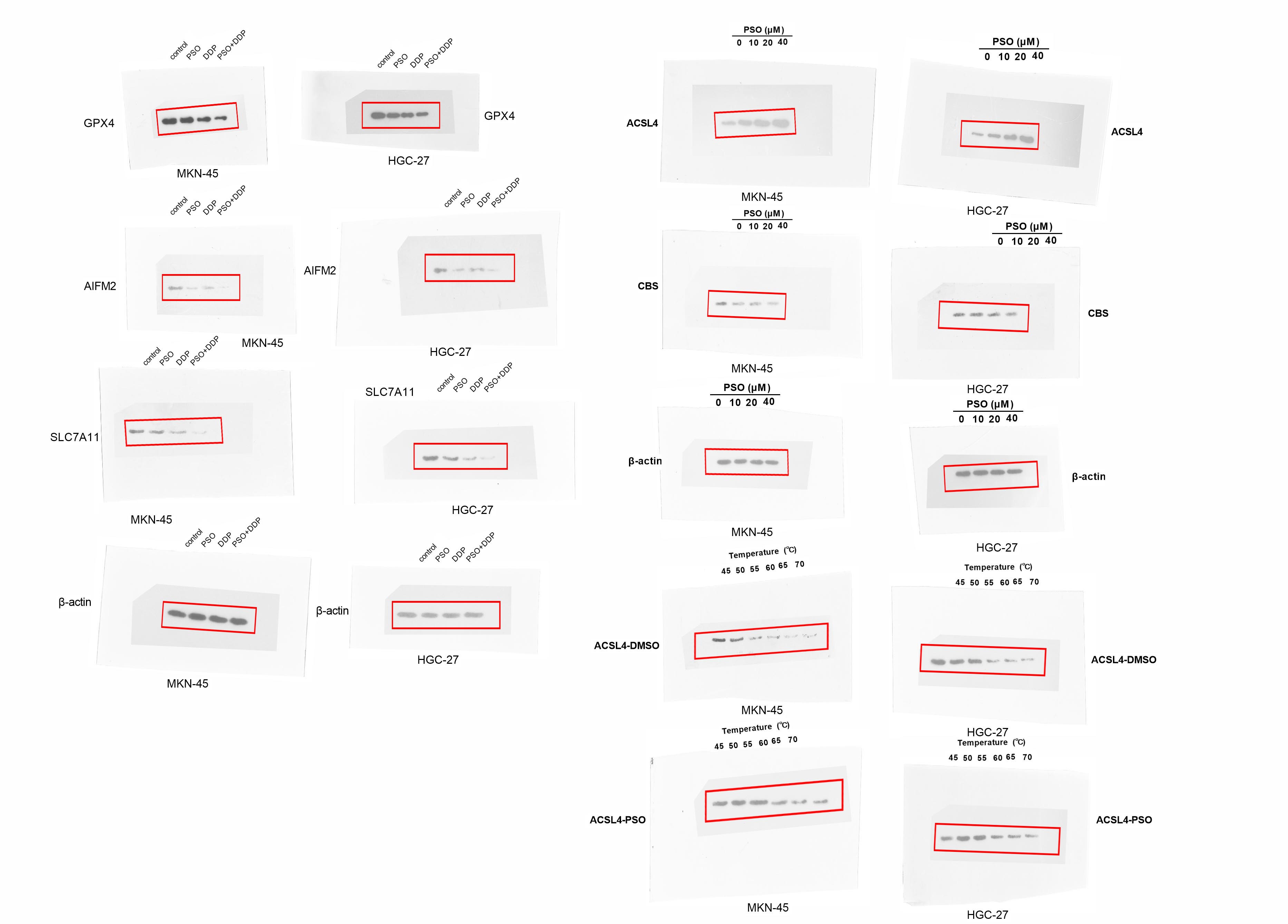

Supplement: Supplementary file 1 — Supplementary Material 1. [file 41065_2025_591_MOESM1_ESM.jpg]
